# Intestinal flora and linear growth in children

**DOI:** 10.3389/fped.2023.1252035

**Published:** 2023-11-16

**Authors:** Pingsihua He, Xingyuan Shen, Sheng Guo

**Affiliations:** Department of Endocrine, Genetics and Metabolism, Shanghai Children’s Hospital, School of Medicine, Shanghai Jiao Tong University, Shanghai, China

**Keywords:** intestinal flora, linear growth, IGF-1 (insulin-like growth factor-1), microbiota, SCFAs (short-chain fatty acids)

## Abstract

The gut microbiota plays a critical role in human growth and development as well as the regulation of human pathophysiological processes. According to research, the gut microbiota controls the host's growth and development in areas such as nutrition, metabolism, endocrine hormones, and immune modulation. The human gut microbiota has an important role in child and adolescent growth, especially when nutritional conditions are poor. In this review, we focus on recent findings about the gut microbiota's influence on child growth, including the relationship between the gut microbiota and linear growth during pregnancy, infancy, childhood, and adolescence. Furthermore, we also review some mechanisms by which intestinal flora influence the host's linear growth. Although the data supports a link between intestinal flora and linear development in children, our review has limitations that prohibit us from fully verifying the causal relationship between gut flora and linear development in children. Improving the gut microbiota, in conjunction with renutrition techniques, has the potential to ameliorate the growth and development impairments currently associated with chronic illness and malnutrition in children.

## Introduction

Human development is divided into four distinct stages: fetal, infant, childhood, and adolescence. Each growth phase is governed by specific endocrine processes and is influenced by genetic, nutritional, and environmental factors (1). Growth retardation is defined as a problem in which individuals have significantly lower growth rates than healthy individuals, which is accompanied by metabolic disorders, systemic inflammation, or intestinal ecological dysregulation (2). After the age of two years, linear growth retardation caused by childhood malnutrition is largely irreversible (3). As a result, prevention or reversal of early interventions for factors contributing to growth retardation provides the best opportunity to improve outcomes (4). Growth retardation is the underlying cause of high morbidity and mortality in children under the age of five (5); it affects approximately 20% of children worldwide (2). Childhood malnutrition, which includes fetal growth restriction, growth retardation, wasting, vitamin A and zinc deficiency, and inadequate breastfeeding, has recently been estimated to cause 3.1 million child deaths per year, accounting for 45% of all child mortality (6). Infants with growth disorders are more likely to die from sepsis, pneumonia, diarrhea, and other infections, as well as from growth retardation (7). Long-term effects of infant malnutrition include short stature and low body weight, immune dysfunction and increased infection risk that persist into adulthood, cognitive impairment, poor academic performance, and decreased productivity in adulthood (8), and being less likely to reach their growth potential in adulthood (3).

## Intestinal flora and human development

The gastrointestinal tract is home to trillions of microbes, known as the “gut microbiome”. Bacteria, fungi, archaea, viruses, and protozoa make up the vast majority of the normal human gut microbiota. Firmicutes and Bacteroidetes were the most abundant in the intestinal flora, while Actinomycetes, Verrucomicrobia, and Proteobacteria were less abundant. The gut microbiota has the potential to influence our physiology in both health and disease (9). Because of the relationship between diet, different physiological states, and the microbiota's ability to produce metabolites from dietary consumption, these important features of the gut microbiota drive research into the functional aspects of microbial diversity (10). Dysbiosis, or disruption of the normal balance between gut microbiota and host, has been linked to the development and progression of obesity and other metabolic disorders, such as diabetes, insulin resistance, and early features of metabolic syndrome, in which the microbiota, the immune system, metabolic pathways, and inflammatory and allergic processes may be involved. Early in infancy, the fecal microbiota is dynamic and can be classified into three separate phases: developmental (3–14 months), transitional (15–30 months), and stable (31–46 months) (9). The first microbial occupancy has a significant impact on the overall health of life. Probiotic supplementation early in life reduces the incidence of neuropsychiatric issues in infancy, according to a study that investigates how the gut microbiota may influence nervous system function (11). Within the first 6 months of life, 75 infants were randomly assigned to either *Lactobacillus* rhamnosus GG or a placebo and were observed for 13 years. At the age of 13, 17.1% of children in the placebo group had attention deficit hyperactivity disorder (ADHD) or Asperger's syndrome, but no children in the probiotic group did (11). Any disruption in the colonization process of the gut microbiome could have long-term consequences for the host's growth, development, and later health. Infants' length, weight, and head and chest circumferences all increase dramatically during this phase of rapid growth. As the gut microbiota matures three years after birth, the prenatal period and the first three years of life are regarded as critical times for the formation of microbial colonization patterns (9). One of the most critical goals of postnatal development is to acquire a gut microbiota capable of benefiting from functions in the environment while also establishing a mucosal immune system capable of tolerating preferred community members and suppressing pathogens. Initially, it was thought that this colonization began during the birth process, but a study describing the identification of bacterial DNA in the placentas of healthy term infants and the discovery of bacteria in the amniotic fluid and meconium of preterm infants confirmed that the fetal gut bacteria groups may have appeared earlier (12).

Many factors influence the development and maturation of the gut microbiota, including placental inflammation, maternal infection during pregnancy, pregnancy course, duration, type of delivery, perinatal conditions, hospital environment and length of stay, feeding methods, antibiotic use, life methods, and geographic factors (13–16). Growth retardation has been linked to a variety of causes, including microbiome dysbiosis, neuroinflammation, endocrine disruptions, starvation, maternal influence, and stress (17). Immature microbiomes interact with risk factors for growth retardation in a two-way fashion, with gut infections, nutrition, birth weight, and other factors both impacting and being influenced by the “growth-restricted” microbiome (2). Overall, improper gut microbiota establishment, particularly in the first two years of life, can affect growth trajectories. Some of the theorized mechanisms of how the gut microbiota influences body weight include increased food energy acquisition, fat deposition promotion, altered exercise activity, satiety effects, and systemic inflammation activation (18).

## Gut microbiota and linear growth

### The influence of gut flora on prenatal linear growth

Fetal growth and development throughout pregnancy are significantly influenced by the fetal environment and the exchange of the fetal-maternal interface. Problems with fetal growth may result from changes in the intestinal flora of the pregnant mother brought on by the mother's genetic make-up, diet throughout pregnancy, delivery method, etc. The findings of genome-wide association studies indicate that there are 12,111 distinct single-nucleotide polymorphisms that are significantly linked with height and are predicted to explain 40%–50% of the phenotypic variance in human height (19). According to studies, the gut microbiota of preterm neonates' siblings in Actinobacteria, Bacilli, Bacteroidia, Clostridia, Erysipelotrichia, and Negativicutes bacteria share significant similarities, implying genetic or shared maternal and environmental effects on the preterm infant gut microbiota (20). Different genetic backgrounds also have an impact on microbial composition, immunological response, and host metabolism, all of which are critical for growth and development. It was shown that some genes associated with growth retardation in genetically defective mice have a dysbiosis of the gut flora. Nod2-deficient mice, for example, lack apoptotic and antifungal responses, have low bacterial populations in the gut, and are vulnerable to pathogenic infections; Card9 knockout mice show hindgut flora dysbiosis (21).

Maternal stunting is a risk factor for low birth weight and subsequent childhood stunting in low- and middle-income countries, sustaining a vicious intergenerational cycle of starvation. This cycle has a negative impact on the child's survival, growth, and neurodevelopment (4). Maternal gut inflammation is linked to poor fetal growth and poor delivery outcomes (4). According to a study of 19 longitudinal birth cohorts, small-for-gestational-age newborns contribute for 20% of childhood stunting and 30% of childhood wasting globally (22). Maternal height was found to be inversely related to child stunting and overall child mortality in all low-income nations (23). This finding could be attributed to physical limits on fetal growth in smaller mothers, but other factors such as maternal inflammation, gut function, microbiota, and epigenetics could also play a role (4, 24). With an estimated 20% of stunting occurring *in utero*, intervention during the first few years of life may not be enough to prevent some of the most severe consequences of growth failure (25).

The diet of a mother during pregnancy and lactation influences the quantity of her microbiota, changing the bacterial repertoire that can be passed down to her kids during pregnancy and early life (26). The acquisition, composition, and microbial activity of the early newborn microbiota are influenced by maternal weight growth during pregnancy. Women who gained more weight during pregnancy had more bacterial diversity and richness than pregnant mothers who gained less weight. Infants born to moms more gestational weight were more likely to have a significant Bacteroidetes pattern and were less likely to have a Firmicutes dominant profile (27, 28). Several studies have confirmed the link between changes in maternal gut flora during childbirth and maternal gestational weight gain. Overweight moms' babies exhibited considerably higher amounts of fecal *Bacteroides* and *Staphylococcus* throughout the first 6 months. More *Bacteroides*, *Clostridium*, and *Staphylococcus* and fewer *Bifidobacterium* were related with higher maternal body weight and body mass index (BMI). Concentrations of *Akkermansia muciniphila*, *Staphylococcus*, and *Clostridium* were lower in infants born to normal-weight moms and mothers who reached normal gestational weight (29, 30).

In utero growth retardation is linked to maternal and placental inflammation and infection, as well as significant changes in host hormone levels, revealing a role for the microbiota-brain axis in fetal growth before birth (31). It is commonly acknowledged that vertical mother-to-infant microbiota transfer has a major impact on baby growth trajectories. Maintaining maternal microbial homeostasis is therefore crucial for preventing metabolic disruptions and growth deficits in children (32). Unfavorable factors, such as an unhealthy maternal diet during pregnancy, can have an effect on the mother's endocrine system and the acquisition of the infant's gut microbiota. One study revealed that a high-fat diet during pregnancy reduced bacterial colonization of the infant's gut and increased enterococci enrichment, with the impact lasting around a month after birth (33).

The manner of delivery, which transmits the neonatal gut microbiota and influences microbial composition, heredity, and function, is a fundamental driver of gut categorization in the first year of life. Vaginal birth has been demonstrated to enhance gut maturation and microbial variety, but cesarean surgery has been linked to gut microbiota acquired through maternal skin commensal bacteria. *Bacteroides* and *Bifidobacterium* were more abundant in infants born vaginally during the first three months of life, *Lactobacillus* and *Bacteroides* during the second three months of life, *Bacteroides* and *Bifidobacterium* during the second six months of life, and *Bacteroides*, *Enterobacter*, and *Streptococcus* after the first year of life. While infants born via cesarean section showed greater levels of *Clostridium* and *Lactobacillus* during the first three months of life, *Enterococcus* and *Clostridium* during the second three months of life, and *Lactobacillus* and *Staphylococcus* beyond the first year of life (34–36).

Preterm delivery, small-for-gestational age (SGA), or both cause approximately 20% of stunting (22). Preterm infants' initial gut microbiota differs from that of full-term newborns, and its microbiota composition is linked to changes in the composition of the mother's gut microbiota. A cross-sectional research of 55 preterm newborns discovered that preterm neonates had much lower gut microbiota alpha diversity and unique beta diversity clustering than term neonates. The contribution of maternal gut microbiota to first preterm gut colonization was greater after spontaneous delivery than after iatrogenic delivery and was not dependent on delivery mode (37). An Italian pilot study discovered that an increase ɑ-diversity levels, and hence a decrease in *Lactobacillus* in the vaginal environment, may be connected with an increased risk of spontaneous preterm birth (38). When compared to full-term infants, the microbiota of preterm infants is determined by the date of gestation, with decreased variety and increased abundance of potentially pathogenic bacteria and decreased abundance of beneficial bacteria such as *Bifidobacterium* (39). SGA infants frequently have difficult pregnancies and deliveries, and prenatal events can alter gut and immune system maturation, as well as impair microbial balance and succession. Furthermore, stressors associated with neonatal life in the hospital, such as frequent antibiotic usage, invasive procedures, and maternal separation, can all lead to dysbiosis (40). A small cohort study discovered that SGA newborns had smaller abundances of *Klebsiella* and *Enterobacter* than AGA infants, and the Beta diversity of bacterial community structure began to segregate at postnatal day 30 (41). Transcriptome investigations of the SGA rat model revealed that IGF-2 expression was considerably reduced in CUG (catch-up growth)-SGA rats, which was associated with a decrease in lactic acid bacteria (42). The gut microbiome influences SGA infants' long-term prognosis in addition to regulating intrauterine growth. The incidence of Neisseriaceae, mucosal-hemolytic bacteria known to absorb iron-bound host proteins including hemoglobin, was considerably greater in the placental microbiota of intrauterine growth restriction (IUGR) patients. Furthermore, the rise of anaerobic bacteria like *Desulfovibrio* represents the development of a hypoxic environment in the IUGR placenta (43). In SGA newborns, certain pathogenic and conditional pathogenic bacteria, such as *Shigella*, *Ralstonia*, and *Clostridium*, increased or became the dominant microbiota. *Bacteroides fragilis* and *Clostridium saccharobutylicum* were detected in SGA newborns and may be linked to neurodevelopmental outcomes at 6 months (44).

In summary, the maternal microbiota is directly linked to the health of the baby, and its disruption can result in fetal growth and development abnormalities such as premature birth, SGA newborns, and macrosomia. Genetic variables (20), nutrition before and during pregnancy (30), manner of birth (36), and gestational age at birth (41) all influence baby microbiome colonization. *Lactobacillus* and *Bifidobacterium* have been shown to promote fetal growth, but pathogenic bacteria such as *Shigella*, *Ralstonia*, and *Clostridium* have been found to inhibit baby growth (29, 39). Furthermore, particular early-life microbes such as *Bacteroides fragilis* and *Clostridium saccharobutylicum* are important for offspring brain development, which can affect baby health and long-term health (44). As a result, sensible treatments to change maternal or offspring microbiome from pregnancy to early childhood have significant implications for offspring health. However, current research on the gut microbiota of mothers and infants is primarily based on 16S RNA gene sequencing results. More research is needed to determine the actual mechanism of the effect on the microbiota and fetal growth, and there is still a long way to go before employing microbiota to interfere in fetal growth. There is still more to be discovered. The investigations on the effect of gut flora on prenatal linear development are summarized in Table 1.

**Table 1:** Summarizes clinical studies on the effect of gut flora on prenatal linear growth.

Clinical situation	Author/year & geographic location	Study design	Growth features & influences	Features of gut microbial community	Identification strategy	PMID
Gestational weight gain	Collado et al., 2010, Finland	Case-control study	Maternal overweight and infant birth weight	The ratio of *Bifidobacterium* to *Clostridium coccoides* was considerably higher in infants born to normal-weight mothers than in infants born to overweight mothers.	FCM-FISH qPCR	20844065
	Stanislawski et al., 2017, Norway	Cohort study	Maternal weight gain, and the gut microbiota	Maternal pre-pregnancy OW/OB and excessive GWG were linked to the highly heritable family *Christensenellaceae*, the genera *Lachnospira*, *Parabacteroides*, *Bifidobacterium*, and *Blautia*.	16S rRNA sequencing	28870230
	Aatsinki et al., 2018, Finland	Cohort study	Gut microbiota and gestational weight gain	Firmicutes-dominated mid-pregnancy showed lower gestational weight growth than Bacteroidetes-dominated mid-pregnancy.	16S rRNA sequencing	29757063
Preterm birth	Prince et al., 2016, United States	Cross-sectional	Placental membrane microbiome and preterm birth	There was a considerable abundance of both urogenital and oral commensal bacteria in preterm patients with chorioamnionitis.	Whole-genome shotgun metagenomics.	26965447
	Jia et al., 2022, China	Cohort study	Preterm infants and gut microbes	The abundance of *Bifidobacterium* rose in the extremely preterm group until 120 days after birth, but it remained steady in the intermediate to late preterm and full term groups from day 14 after birth.	16S rRNA sequencing	35847070
	Chu et al., 2016, United States	Cohort study	Maternal high-fat diet and infant gut microbiome varies	*Bacteroides* levels were significantly lower in neonates exposed to a high-fat pregnancy diet.	16S rRNA sequencing	27503374
	Tirone et al., 2022, Italy	Pilot study	Maternal and neonatal microbiota and spontaneous preterm birth	Decrease in *Lactobacillus* in the vaginal environment, may be connected with an increased risk of spontaneous Preterm Birth	16S rRNA sequencing	35935374
	Hiltunen et al., 2022, Finland	Cross-sectional study	Maternal and neonatal microbiota and preterm birth	Preterm neonates had much lower gut microbiota alpha diversity and unique beta diversity clustering than term neonates.	16S rRNA sequencing	34349229
Small-for-gestational age	Chang et al., 2022, Taiwan	Cohort study	Gut microbiota and very low birth weight	SGA newborns had smaller abundances of *Klebsiella* and *Enterobacter* than AGA infants.	16S rRNA sequencing	36501188
	Chen et al., 2022, China	Cohort study	Gut microbiota in SGA infants and neurodevelopmental outcomes	Bacteroidota, *Bacteroides*, *Bacteroides fragilis*, and *Clostridium saccharobutylicum* may be linked to neurodevelopmental outcomes of SGA infants.	16S rRNA sequencing	36090083
	Hu et al., 2021, United States	Pilot study	Placental microbiota and SGA	The prevalence of *Neisseriaceae*, mucosal-hemolytic bacteria, was significantly higher in IUGR patients’ placental microbiome.	16S rRNA sequencing	33107014

FCM-FISH, fluorescence *in situ* hybridization combined with flow cytometry; qPCRs, quantitative real-time PCRs; OW/OB, overweight/obese, GWG, gestational weight gain; SGA, small-for-gestational age.

### Intestinal flora and early infant growth and development

Colonization of the newborn gut is thought to be vital for healthy growth because it influences gut maturation, metabolic, immunological, and brain development in early life. Microbiota interactions throughout infancy may be an important predictor of the host's long-term metabolic effects (45). Infants' early eating patterns and nutritional status are critical for the early molding of intestinal flora, and the interplay between intestinal flora and nutrition is critical for growth and development during infancy. In southern India, a longitudinal investigation of the gut microbiota of 10 infants with low birthweight and chronic stunting and 10 children with normal birthweight and no indications of stunting was done. From 3 months to 24 months of age, fecal samples were collected and examined every 3 months. The LEfSe algorithm was used to analyze differentially enriched taxa and found that the microbiota of stunted children was enriched in inflammatory bacteria from the Proteobacteria phylum, whereas the microbiota of normally developing children was enriched in probiotics, such as *Bifidobacteria longum* (12). A 6-year retrospective study of preterm children born at 35 weeks indicated that optimum postnatal nutrition enhanced early catch-up weight growth and improved linear development while having no influence on childhood BMI (46). According to a research of 108 healthy neonates in their first half year of life, breastfed newborns had more *Lactobacillus*, *Bacteroides*, and *Bifidobacterium* and less pathogens in their gut, which correlates to an accelerated rate of growth in infants (45). A significant decrease in the abundance of sialylated human milk oligosaccharides (HMOs) in human milk can result in severe growth failure in infants. Supplementation with sialylated HMOs promotes microbiota-dependent growth in stunted infants, possibly due to increased *Bifidobacteriaceae* abundance in infant gut (47). The amount of this oligosaccharide in the milk of malnourished mothers was reduced. The researchers discovered increased muscle mass, stronger bones, and significant changes in liver and brain metabolism when they administered oligosaccharides purified from whey to mice transplanted with stool from severely malnourished children. It implies that these findings have far-reaching implications and that controlling intestinal flora can affect children's nutritional status, but more clinical research is needed to back up these animal-based speculations (48). Microbial changes caused by weaning disruption delay intestinal barrier maturation and increase susceptibility to allergic inflammation, which may result in later growth retardation (49). Human breast milk contains a “different type of lactose” than cow's milk, which contains hundreds of oligosaccharides that promote Bifidobacteriales growth, according to research. Breastfeeding promotes the development of sensible and beneficial flora and assists babies in developing normally (50).

Poor infant hygiene and antibiotic-induced intestinal flora disruption are other key causes of linear growth disorder in newborns and early children. In Bangladeshi 2-years-old, small intestinal bacterial overgrowth (SIBO) was linked to poor hygiene, intestinal inflammation, and shorter length for age (51). Although no differences in intestinal permeability fecal markers were found in these SIBO-positive children, they did have elevated fecal calprotectin levels and were more likely to have growth retardation by the age of two (52). Evidence suggests that early-life antibiotic exposure is related to baby development and speed. One putative biological mechanism underpinning the effects of antibiotics on offspring development was structural and functional changes in the gut microbiota. A study from the Shanghai Mother-Child Pair Cohort examined 18 common antibiotics in meconium, including chlortetracycline, penicillin, and chloramphenicol, and used a multivariate linear regression model to examine antibiotic exposure, infant gut flora, and growth and development. Interdependence of indicators penicillin was discovered to have a negative relationship with gut microbiota Pielou and Simpson's index and a favorable relationship with growth velocity at 2–6 months (53). Another research investigating the long-term effects of neonatal and early childhood antibiotic exposure on child growth in an unselected birth cohort of 12,422 full-term infants discovered that males had significantly lower weight and height gains than girls during the first 6 years of life. Neonatal antibiotic exposure was linked to significant changes in the gut microbiome, notably a decrease in the number and diversity of fecal Bifidobacteriales before the age of two. Transplanting fecal microbiota from antibiotic-exposed children onto germ-free male mice resulted in substantial growth failure. Antibiotic exposure during pregnancy has been related to long-term changes in the gut microbiota, which may result in reduced growth in males during the first six years of life, according to this research (54). Another study revealed that early antibiotic exposure was not connected with enhanced growth velocity between delivery and discharge in neonates and infants in intensive care units inpatient antibiotics (55).

Linear growth disorder in infancy is connected with abnormal immunological inflammation and the disruption of gut flora. Prior to growth decrease, children with developmental delay had higher gut bacterial diversity and elevated inflammatory biomarkers, according to a longitudinal study of 78 Peruvian infants aged 5–12 months. Throughout the study, the fecal microbiota composition of stunted children was more diverse than that of healthy controls. *Ruminococcus 1* and *2*, *Clostridium sensu stricto*, and *Collinsella* abundance increased in stunted children but not in controls, but *Providencia* abundance dropped. The authors suggest that chronic, low levels of microbial translocation across the gastrointestinal mucosa may be the source of immune activation in children with developmental delays. However, because abnormalities in the gut microbiome exist prior to growth retardation, inflammatory chemicals derived by microbes may also contribute to chronic local irritation (56). Another study of 46 duodenal samples, 57 stomach samples, and 404 stool samples from stunted children aged 2–5 years in Africa found that the vast majority of stunted children exhibited gastrointestinal symptoms. In addition, *Escherichia coli/Shigella* sp. and *Campylobacter* sp. were shown to be more common in stunted children, although Clostridia, well-known butyrate makers, were reduced in comparison to nonstunted children. Oral bacteria were overrepresented in fecal samples from stunted children (57).

During infancy, there is a “critical window” for gut microbiota development, and disruptions in this process may be critical for children's growth and development (9, 16). Nutritional status (46), feeding techniques (47), hygienic conditions (51), antibiotic use (53), and intestinal inflammation (56) are major factors that induce intestinal flora alteration and impair children's growth and development, according to current clinical research. The majority of research support the identification of gut flora, such as *Bifidobacterium*, *Bacteroides*, and *Lactobacillus*, as being favorably associated with early baby growth. Actinomycetes, particularly Bifidobacteriales, are the most prevalent members of the gut microbiota in well-growing newborns (45, 47). However, research reports on the intestinal flora related with infant development retardation differ widely, owing to overgrowth of intestinal flora, overexpression of pathogenic bacteria, an abundance of *Ruminococcus*, *Clostridium sensu stricto*, and *Collinsella*, among other factors (51, 56). This may be related to the various research objects chosen by the researchers. In short, infancy is a vital stage for the creation of gut flora. Food influences the gut flora. The connection between nutritional status and gut flora is a significant area of interest in baby growth and development research. The clinical investigations on the effect of gut flora on early infant growth and development are summarized in Table 2.

**Table 2:** Summarizes clinical studies on the effect of gut flora on early infant growth and development.

Clinical situation	Author/year & geographic location	Study design	Growth features & influences	Features of gut microbial community	Identification strategy	PMID
Eating patterns and nutritional status	Tadros et al., 2022, United States.	Retrospective study	Nutritional support and microbiome acquisition.	Gammaproteobacteria postnatal abundance was inversely associated with early growth, but *Bacteroides* and *Lactobacillus* were favourably associated with childhood BMI.	16S RNA V4 sequencing	36016882
	Martin et al., 2016, Netherlands	Observational study	Nutritional supplementation and infant gut microbiome development	The presence of *Bifidobacterium animalis subsp. lactis* was discovered to be completely dependent on the method of feeding.	qPCR/RT qPCR	27362264
	Berger et al., 2020, Switzerland	Randomized controlled trial	Human milk oligosaccharides and infant fecal community	Supplementing newborns with sialylated HMOs enhances microbiota-dependent growth, presumably due to increased *Bifidobacteriaceae* abundance in the infant gut.	16S RNA V3–V4 sequencing	32184252
	Charbonneau et al., 2016, United States	Cohort and experiment study	Human milk oligosaccharides and infant fecal community	S-BMO promotes growth in gnotobiotic mice harboring the malawian infant culture collection and fed a prototypic malawian diet.	16S rRNA sequencing and COPRO-seq	26898329
Poor infant hygiene and antibiotic-induced intestinal flora disruption	Donowitz et al., 2016, United States	Cross-sectional study	Small intestinal bacterial overgrowth and shorter length	The markers of intestinal inflammation fecal Reg 1β and fecal calprotectin were elevated in SIBO-positive children.	Gas chromatograph	26758185
	Zhou et al., 2021, China	Cohort study	Antibiotic exposure, neonatal gut bacteria and infant growth speed	Penicillin exposure has a negative link with gut microbiota Pielou and Simpson's index and a positive relationship with growth velocity at 2–6 months.	16S RNA V3–V4 sequencing	34371267
	Uzan-Yulzari et al., 2021, Israel and Finland	Birth cohort study	Antibiotic exposure, neonatal gut bacteria and infant growth speed	Antibiotic therapy had the greatest impact on the genus *Bifidobacterium*.	16S RNA V4 sequencing and metagenomics	33500411
	Reid et al., 2019, United States	Retrospective study	Neonatal antibiotic exposure and infant growth	Antibiotic exposure was not connected with growth velocity between delivery and discharge in neonates and infants in ICU.	–	30500965
Gut inflamation and disruption of flora	Zambruni et al., 2019, United States	Pilot prospective study	Linear growth, intestinal damage, and systemic inflammation	*Ruminococcus* 1 and 2, *Clostridium sensu stricto*, and *Collinsella* abundance increased in stunted children but not in controls, but *Providencia* abundance dropped.	16S RNA V4 sequencing	31482782
	Vonaesch et al., 2018, France	Clinical trial	Microbiome “decompartmentalization” and linear growth delay	Oral bacteria were found to be overrepresented in fecal samples from stunted children, as were *Escherichia coli/Shigella* sp. and *Campylobacter* sp., while Clostridia, well-known butyrate makers, were reduced.	16S RNA sequencing and semiquantitative culture methods	30126990

BMI, body mass index; COPRO-Seq, COmmunity PROfiling by sequencing; HMOs, human milk oligosaccharides; S-BMO, sialylated bovine milk oligosaccharides; SIBO, small intestine bacterial overgrowth.

### Adolescent linear growth and intestinal flora

Adolescence is a transitional period between childhood and maturity, a period of physical, neurological, psychological, and social changes, and a key time for growth and development. Some chronic mental illness states in childhood and adolescence, such as cognitive, emotional/social disturbances, sensory functional impairment, communication impairment, and so on, are intimately related to adolescents' growth and development. Although the pathophysiology of how psychiatric diseases such as anorexia nervosa (AN), ADHD, and autism spectrum disorder (ASD) affect growth and development in adolescents is not fully known, dysbiosis of the microbiota has been proposed as a possible explanation (18). AN is a severe psychiatric condition that primarily affects adolescents as a result of the severely detrimental consequences of caloric restriction on linear growth during puberty (58). A study of the composition and diversity of the gut microbiome in adolescents with anorexia before and after nutritional supplementation discovered that patients had greater individual differences in gut bacterial and metagenomic content, with fecal levels of serotonin, gamma-aminobutyric acid, dopamine, butyrate, and acetate decreasing in the samples (59). ADHD is one of the most frequent neurodevelopmental diseases in children. Numerous studies indicate that ADHD is linked to teenage growth and development (60). A Finnish study revealed that adolescents with hyperactive-impulsive ADHD were taller, and elementary school kids with ADHD were shorter and smaller than a control group of children of the same age (61). A case-control research comparing Chinese children with ADHD to healthy controls discovered that those with ADHD had lower levels of *Faecalibacterium* and *Veillonellaceae*, whereas *Enterococcus* and *Odoribacter* were significantly higher (62). Despite the fact that autistic children's gut microbiota differs significantly from that of normal children, with a reduced proportion of *Coprococcus* and *Bifidobacterium*, the children's height does not alter (63). In adolescence, there has been minimal research on height in children with ASD, and some studies have found that the ASD group was significantly larger than the control group in terms of head circumference, weight, and BMI, but there was no difference in height (64).

Nutritional status can influence linear bone growth during adolescence and puberty by regulating growth plate chondrocytes, which are critical components of juvenile growth (65). Excess body weight early in childhood might have an impact on growth patterns. There is evidence that excess adiposity throughout childhood alters growth patterns and pubertal development. Several studies have found that obese children have a higher height velocity and a faster bone age during their prepubertal years (66). Several hormones released by adipose tissue may influence linear growth in the context of obesity, both through the growth hormone insulin-like growth factor-1 (IGF-1) axis and directly through the epiphyseal growth plate. In 11 of the 12 cohort studies that were the subject of a systematic review and meta-analysis, there was a positive connection between total protein intake and BMI. The meta-analysis suggested a favorable relationship between total protein intake and BMI. There may also be evidence showing a connection between a higher intake of animal protein in the diet and a higher BMI. However, there is no clear evidence linking total protein intake with an elevated risk of being overweight or obese. Only suggestive data partially support this association between total protein intake and an elevated risk of being overweight or obese (67). The effect of nutrition on the gut flora during adolescence is linked to linear growth in puberty. In respect to linear growth, a study of 350 girls aged 12–13 years that followed three major dietary patterns: healthy, heavy in sugar and salt, and a Western diet found that a healthy dietary pattern with enough intake of plant protein and white meat was connected with more favorable linear growth (65). Diet and dietary components have a significant impact on the composition of the gut microbiota and are among the most important contributors to bacterial flora changes. Existing research suggests that adopting a plant-based diet benefits the host microbiome, reduces inflammation, improves insulin sensitivity, and promotes optimal energy balance, which can lead to the prevention of chronic low-inflammation-related diseases (68).

In comparison to the high prevalence of malnutrition in newborns and early children, obesity or overweight in adolescents has a greater influence on children's growth and development. Obesity with developmental delay is more common in younger children and adolescents than obesity without developmental delay. A research in Vietnam found that 5% of overweight children were also stunted, whereas a study in Sao Paulo, Brazil found that 6% of children in low-income urban families were overweight and stunted, and that obesity with stunting was more common than obesity without stunting. Similarly, growth retardation and increased obesity were shown to coexist in a study of young children in urban areas of the Cape Peninsula, South Africa, and researchers believe that the community has transitioned from undernutrition to overnutrition without reaching optimal nutritional status (69). A bioinformatically re-analyzed study based on published amplicon sequencing data from the National Center for Biotechnology Information discovered that the impacts of obesity on the gut microbiota may be more severe in infancy than in adulthood and eventually endure throughout life. The study discovered significant changes in gut microbiota between children with and without obesity, while no similar differences were seen in the adult group. Using gut microbiota to predict pediatric obesity is more difficult, according to random forest models, than adult obesity. The data show that the gut microbiota is more vulnerable in childhood than in maturity (70). A cross-sectional study of taxonomic characteristics of the gut microbiota in 46 children and their association with obesity in diet-dependent children discovered that children with an abundance of *Holdemania* spp. and high protein and complex carbohydrate consumption had a lower z-BMI, waist circumference, and hip circumference. In contrast, they found a link between *Coprococcus catus* and a low intake of this dietary pattern and hip circumference (71). Decades of observational research have revealed differences in the composition of the gut microbial community between obese and healthy people, and seminal studies in which fecal microbes from obese adults were transplanted into gnotobiotic mice recapitulate weight gain and obesity-related metabolic signatures, demonstrating a direct causal link between disrupted gut microbiota and obesity. Correction of flora issues appears to help prevent or treat obesity-related growth and metabolic disorders (72). However, clinical trials of microbiota-targeting treatments have had conflicting outcomes. A recent systematic review and meta-analysis (73) identified 19 trials comparing probiotics or synbiotics to any strategy other than bariatric surgery or FMT. Individual trials show no significant improvement in body weight, while combined case analyses show no differences in body weight or body mass index between probiotics or synbiotics and controls (73). Obese children mature more quickly than lean children, which increases the risk of poor adult height and early puberty. Despite the fact that obese children have a quicker linear growth rate, body obesity may promote neuroendocrine events that contribute to the start of puberty (74).

Precocious puberty, which can disrupt the gut microbiome, is another concern associated with linear growth in teenagers. According to a Korean study that investigated the composition of the gut microbial community in obese teenagers, the abundance of *Bacteroides* and *Prevotella* is strongly associated with BMI, and the composition of *Bacteroides* is adversely associated with triglycerides and total cholesterol (75). Dong et al. (76). discovered changes in the gut microbiota between individuals with idiopathic central precocious puberty (ICPP) and healthy girls. They discovered that the gastrointestinal genera found in ICPP are comparable to those linked to obesity, including *Ruminococcus*, *Gemmiger*, *Oscillibacter*, and *Clostridium XIVb*. In terms of microbial species, girls with ICPP had higher amounts of *Rumicoccus bromii*, *Ruminococcus gnavus*, and *Ruminococcus leptum*. The first two were discovered in obese people and were found to boost energy absorption and adipose tissue hyperplasia, whereas *Ruminococcus leptum* was found to influence human weight changes. These findings underscore the link between obesity, ICPP, and gut microbiome dysbiosis (32, 77). Another study discovered that the gut flora of central precocious puberty patients behaves similarly to that of other neurological illnesses, with an abundance of *Alistipes*, *Klebsiella*, and *Sutterella*. These microbes create neurotransmitter-like metabolites (serotonin and dopamine), which initiate early puberty and activate the hypothalamic-pituitary-gonadal axis (78). Although accurate estimates of height loss due to premature puberty are difficult to obtain, prior studies of untreated patients revealed an average height loss of 10 cm in girls and 20 cm in boys. Girls who receive gonadotropin-releasing hormone analog (GnRHa) treatment before the age of 6 years, on the other hand, can achieve a final height gain of 2–10 cm (79, 80). When compared to Tanner stage-matched controls, girls with true central precocious puberty show an adverse metabolic profile at diagnosis; even GnRHa treatment cannot correct this shortfall (81). The investigations on the effect of gut flora on adolescent linear growth are summarized in Table 3.

**Table 3:** Summarizes studies on the effect of gut flora on adolescent linear growth.

Clinical situation	Author/year & geographic location	Study design	Growth features & influences	Features of gut microbial community	Identification strategy	PMID
Psychiatric diseases	Prochazkova et al., 2021, Czech Republic	Longitudinal study	Anorexia nervosa and intestinal microbiota	*Alistipes*, Clostridiales, *Christensenellaceae*, and *Ruminococcaceae* were the most common taxonomically distinctive flora in patients with AN.	16S rRNA sequencing and ITS7/ITS4 sequencing	33779487
	Wan et al., 2020, China	Case-control study	ADHD and intestinal microbiota	In the ADHD group, *Faecalibacterium prausnitzii*, *Lachnospiraceae bacterium*, and *Ruminococcus gnavus* were greatly reduced, but *Bacteroides caccae*, *Odoribacter splanchnicus*, *Paraprevotella xylaniphila*, and *Veillonella parvula* were significantly increased.	Shotgun metagenomic sequencing	32132899
	Iglesias-Vazquez et al., 2020, Spain	Systematic review and meta-analysis	ASD and intestinal microbiota	Children with ASD showed a significantly higher abundance of the genera *Bacteroides*, *Parabacteroides*, *Clostridium*, *Faecalibacterium*, and *Phascolarctobacterium* and a lower percentage of *Coprococcus* and *Bifidobacterium*.	16S rRNA sequencing, bacterial tag encoded FLX amplicon pyrosequencing, PCR or culture	32192218
Nutritional status	Yu et al., 2023, China	BioData mining	Obesity and gut microbiota	The gut microbiota was more vulnerable in childhood than in maturity	16S rRNA sequencing	36776907
	Orbe-Orihuela et al., 2022, México	Cross-sectional study	Diet-dependent childhood obesity and gut microbiota	Children with an abundance of *Holdemania* spp. and high protein and complex carbohydrate consumption had a lower z-BMI, waist circumference, and hip circumference.	Whole metagenome shotgun sequencing	35382951
	Suzumura et al., 2019, Brazil	Meta-analysis	probiotics or synbiotics and overweight	Although the quality of evidence is low to moderate, oral supplementation with probiotics or synbiotics has a slight effect on waist circumference but no effect on body weight or BMI.	Commercial probiotic powder, commercial probiotic yogurt, commercial synbiotic capsules or probiotic edam-type cheese	30924853
Precocious puberty	Dong et al., 2019, China	Cross-sectional study	Precocious puberty and gut microbiota	The ICPP group had higher GM diversity and was enriched for several GM species, including *Ruminococcus gnavus*, *Ruminococcus callidus*, *Ruminococcus bromii*, *Roseburia inulinivorans*, *Coprococcus eutactus*, *Clostridium leptum*, and *Clostridium lactatifermentans*, all of which are linked to obesity and the production of short-chain fatty acids.	16S rRNA sequencing	32038493
	Li et al., 2021, China	Cross-sectional study	Precocious puberty and gut microbiota	CPP patients have an abundance of *Alistipes*, *Klebsiella*, and *Sutterella*, which is comparable to other neurological disorders.	16S rRNA sequencing	33634977

ADHD, attention-deficit/hyperactivity disorder; AN, anorexia nervosa; ASD, autism spectrum disorder, CPP, central precocious puberty; GM, gut microbiota; ICPP, idiopathic central precocious puberty.

### Key bacterial taxa associated with linear growth at different stages of childhood

**Figure 1:**
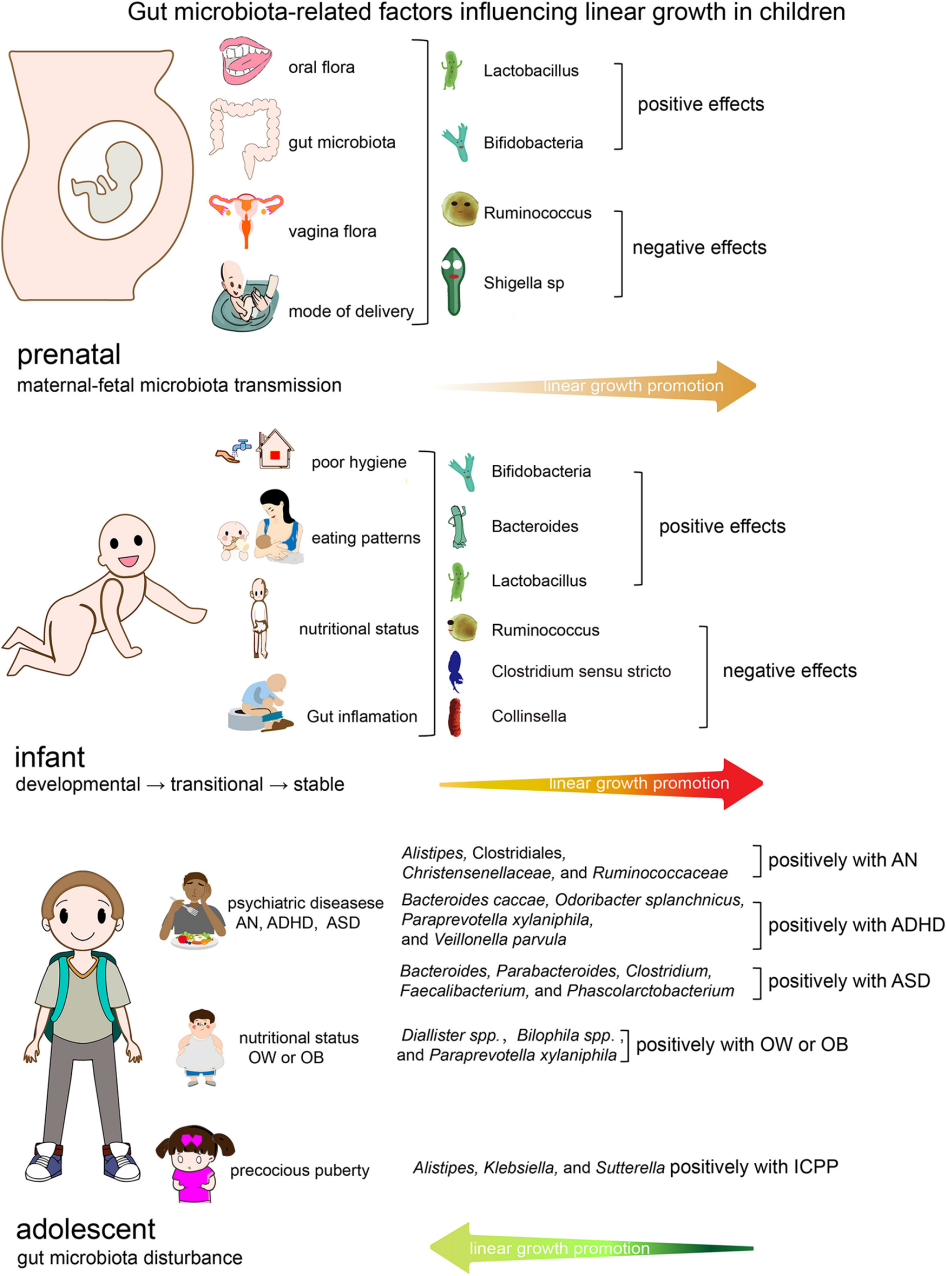
Gut microbiota-related factors influencing linear growth in children. OW/OB, overweight/obese; ADHD, attention-deficit/hyperactivity disorder; AN, anorexia nervosa; ASD, Autism spectrum disorder.

Evidence suggests that certain aspects of the gut microbiota are linked to certain stages of growth and development in children (18). Figure 1 depicts the characteristics of intestinal flora at various phases of children's growth and development, as well as the impact of intestinal flora on linear growth in children. The oral cavity (31, 82), vagina (36), and intestines (37) of the mother are major sources of neonatal intestinal flora. Hematogenous transfer of maternal oral bacteria during pregnancy may be an important factor in placental colonization (31, 83). Evidence suggests that patients who gave birth prematurely had a distinct gut microbiome dysbiosis compared to those who gave birth at term. *Porphyromonas*, *Streptococcus*, *Fusobacterium*, and *Veillonell* a were enriched in the preterm group, whereas *Coprococcus* and *Gemmiger* were significantly depleted. The majority of the enriched bacteria were oral bacteria that had been annotated (82). The mother's gut and the maternal vagina are the principal sources of microbiota for vaginally delivered newborns (84). The microbiome of newborns born via cesarean section is mostly derived from the mother's skin and the hospital environment (85). The most important factors influencing prenatal growth and development are prenatal weight gain (28–30), premature birth (31, 38, 39), and being small for gestational age (41, 44). According to research, these elements are linked to the creation and features of newborn gut flora. As illustrated in Figure 1, *Lactobacillus* and *Bifidobacterium* are advantageous to newborn growth throughout the perinatal period, however pathogenic bacteria such as *Shigella*, *Ralstonia*, and *Clostridium* may be detrimental. During infancy, nutritional status, feeding procedures, hygienic conditions, antibiotic usage, and intestinal inflammation are important factors that affect intestinal flora and impede children's growth and development. As illustrated in Figure 1, gut flora such as *Bifidobacterium*, *Bacteroides*, and *Lactobacillus* are favorably associated with infant growth. Pathogenic bacterial overexpression, as well as an abundance of *Ruminococcus*, *Clostridium sensu stricto*, and *Collinsella*, are all unfavorable factors. Infants' gut flora has stabilized between 31 and 46 months, hence children's growth rate tends to be constant and relatively slow from infancy until pre-adolescence (9). Adolescence is characterized by a succession of physical, neurological, psychological, and social changes, as well as the second growth spurt in life. Microbiota abnormalities associated with children's growth and development during this period are mostly associated with some particular diseases such as AN (59), ADHD (62), ASD (63), precocious puberty (76, 78), and obesity (71). *Alistipes*, Clostridiales, *Christensenellaceae*, and *Ruminococcaceae* abundance was linked to AN (59). ADHD was linked to an abundance of *Bacteroides caccae*, *Odoribacter splanchnicus*, *Paraprevotella xylaniphila*, and *Veillonella parvula* (62). *Bacteroides*, *Parabacteroides*, *Clostridium*, *Faecalibacterium*, and *Phascolarctobacterium* are more abundant in the gut of ASD children (63).

### Possible mechanism of gut microbiota affecting linear growth

The anterior pituitary gland secretes growth hormone, which regulates insulin-like growth factor-1 (IGF-1) synthesis. One of the most fascinating theories that could explain the association between gut microbiota composition and linear and bulky growth in children is the involvement of gut microorganisms in boosting growth hormone. The gut microbiota can influence linear growth by influencing growth axis activity and changing hormone release. It could be one of the key methods by which the gut flora regulates linear development by modulating the action of the GH (growth hormone)/IGF-1 axis. Children who have chronic nutritional deficiencies develop growth hormone resistance and become stunted. The liver and peripheral tissues, including muscle, create IGF-1 which promotes growth throughout the body and organs. IGF-1 is a key mediator of bone growth that works in the endocrine, paracrine, and autocrine systems (86). In one mice investigation, researchers demonstrated that *Lactobacillus plantarum* strains in the gut microbiota maintain growth hormone action via signaling pathways in the liver, overcoming malnutrition-induced GH resistance. According to this research, certain beneficial bacteria can be employed to treat stunted growth and development caused by malnutrition (87). A study of Drosophila revealed that a common bacterium, *Acetobacter pomorum*, can modulate insulin/insulin-like growth factor signaling and hence influence Drosophila developmental speed, body size, and energy metabolism (88). On the one hand, excess short-chain fatty acids (SCFAs) produced by a specific benign bacteria provide an additional source of energy, resulting in an imbalance in energy regulation that promotes growth. SCFAs, on the other hand, have a role in glucose-stimulated insulin secretion from cells through interactions with the free fatty acid receptors FFA2 and FFA3, as well as the release of hunger-regulating peptide hormones. This seemingly contradictory finding demonstrates that, in addition to the SCFA levels produced by some gut microbiota members, some G-protein-coupled receptors (GPCRs) in the host can directly identify specific bacterial components, thereby controlling other metabolic processes that may contribute to body growth (10). In obese or overweight patients, a 12-week intervention with a low-carbohydrate diet (LCD) resulted in a considerable increase in the relative abundance of butyrate-producing bacteria, including *Parabacteroides* and *Oscillospira*. Furthermore, in the LCD group, participants with higher relative abundance of *Bacteroides* at baseline responded better to the LCD intervention and had greater weight loss outcomes. Some *Oscillospira* species may produce considerable levels of SCFAs, which aid in weight management as well as glucose and lipid homeostasis. Another notion is that *Oscillospira* can disrupt host glycans, allowing hosts to expend metabolic energy to restore destroyed glycoproteins. In this study, the increased abundance of *Parabacteroides* and *Oscillospira* could be a gut microbiota response to dietary intervention, assisting in LCD weight loss (89). Children who grow up in polluted surroundings are more likely to get intestinal infections and malnutrition, and they are more likely to develop overweight/obesity and accompanying comorbidities later in life. When exposed to energy-rich meals, children who had height deficits in childhood were more likely to be overweight or obese as adults (90). To summarize, numerous studies have demonstrated that gut flora is closely associated to obesity, and that changing gut flora may be an essential factor in weight control. A randomized clinical trial of adolescent obesity patients discovered that fecal microbiota transplantation (FMT) had no effect on weight loss but did ameliorate metabolic abnormalities (91). The most important link between and host IGF-1 levels appears to be SCFAs metabolized by gut bacteria from indigestible fiber-rich diets, but other mechanisms by which gut microbes influence bone growth may exist (92).

Another potentially growth-promoting mechanism for gut bacteria is the tryptophan (TRP)-kynurenine (KYN) -niacin pathway. In this process, indoleamine 2,3-dioxygenase (IDO) converts dietary tryptophan to kynurenine, which is then converted to niacin. Niacin is an important precursor of nicotinamide adenine dinucleotide (NAD+). Children with a greater kynurenine to tryptophan ratio (KT) had lower linear growth, according to studies, and experimental animal models have also indicated that a tryptophan deficit is associated with decreased growth velocity (93, 94). Undernutrition was found to affect various metabolic pathways, including choline and tryptophan metabolism, while also increasing the proteolytic activity of the gut flora. Additionally, metabolic adaptation to lower energy expenditure was observed in malnourished children, as demonstrated by increased N-methylnicotinamide and decreased -aminoisobutyric acid excretion. Undernourished children with stronger metabolic adaptability demonstrated quick catch-up growth many months early (95). The KYN: TRP/ KT ratios were shown to be strongly linked with children's growth in a study of stunted children in Bangladesh. This change in the TRP pathway could be due to environmental stresses such as chronic inflammation, environmental enteric dysfunction, inadequate protein consumption, or alterations in the gut microbiota, all of which can have a substantial impact on growth (96).

Furthermore, Gut microbiota may prompt immune cells to release certain cytokines, influencing the host's linear growth (56, 97, 98), and greater intestinal permeability produced by environmental intestinal dysfunction is hypothesized to cause higher bacterial product translocation, culminating in systemic inflammation and immunological activation (99). Chronic inflammatory illnesses, such as inflammatory bowel disease and juvenile idiopathic arthritis, are a major cause of stunted growth and development in children (100, 101). Systemic inflammation and immune activation may play a role in the development of linear growth disorders via cytokine-induced anorexia, nutrient utilization regulation, and/or interference with the growth hormone axis and bone metabolism (99). In a Zimbabwe birth cohort study, Prendergast et al. discovered that developmental delay was related to raised systemic inflammatory markers (C-reactive protein and 1-acid glycoprotein) and lower IGF-1 levels. The presence of intestinal injury in infants with developmental delays was demonstrated by higher plasma intestinal fatty acid-binding protein levels (102). Saari et al. discovered that children with *Helicobacter pylori* infection had low serum acylated ghrelin and growth retardation in a one-year longitudinal cohort study. *Helicobacter pylori* eradication successfully restores ghrelin levels and increases growth in children (103). Saari et al. found the same link between antibiotics and height in a large cohort of Finnish infants (104), and a meta-analysis of 10 randomized controlled trials found that antibiotic use increased height by 0.04 cm/month (105). However, in Niger, a large-scale study of azithromycin use and growth and development in children aged 6–60 months noticed no link between antibiotic use and improved human growth (106). Kosek et al. observed that kids with higher levels of fecal biomarkers of intestinal inflammation and intestinal barrier disruption were more likely to have linear growth retardation during the first 18 months of life in an international multicenter prospective study (107). Current studies (17, 104–107) on antibiotics and children's growth vary significantly, and there are many reasons for some of the differences, but the influence of environmental factors, which include the nutritional status of the children included in the studies, hygiene, feeding practices, drinking water, chronic inflammatory diseases, endocrine disorders, and so on, must all be considered.

**Figure 2:**
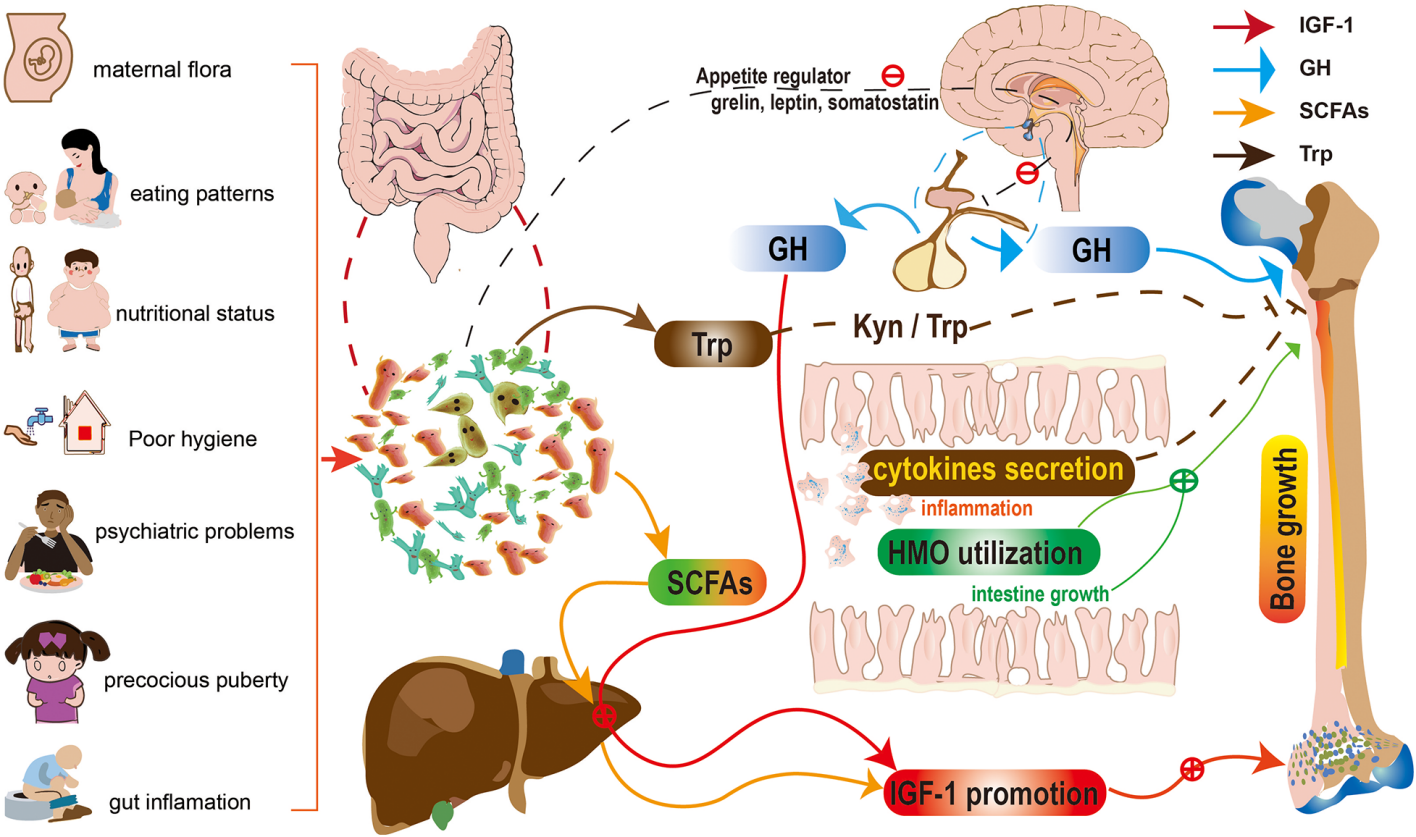
Schematic representation for the role of gut microbiota in children's linear growth. The potential mechanisms include: (1) alterations in food absorption (i.e., increased levels of health-promoting microbial metabolites such SCFAs and HMOs); and (2) endocrine hormones (GH/IGF-1 axis). (3) Changes in immune regulation (including cytokines and gut inflammation); (4) Control of the gut-brain-bone axis (including neurotransmitters, appetite control, and Kyn/Trp). HMOs, human milk oligosaccharides; SCFAs, Short-chain fatty acids; Kyn/Trp, kynurenine to tryptophan ratio; GH, growth hormone; IGF-1 insulin-like growth factor 1.

In summary, the determinants of stunting operate at multiple causal levels, ranging from the most distal socioeconomic and political variables to the most proximal, such as food quantity and quality, as well as their biotransformation by the gut microbiota, host infection, immune dysfunction, and systemic physiology (108). Figure 2 shows a schematic representation of the gut microbiota's contribution to children's linear growth. The potential mechanisms include: (1) alterations in food absorption (10, 47) (i.e., increased levels of health-promoting microbial metabolites such SCFAs and HMOs); and (2) endocrine hormones (86) (GH/IGF-1 axis). (3) Changes in immune regulation (56, 97) (including cytokines and gut inflammation); (4) Control of the gut-brain-bone axis (94, 96) (including neurotransmitters, appetite control, and Kyn/Trp).

## Conclusion and future perspectives

Microbiome research is still in its early stages and has mostly concentrated on bacterial taxa, but the microbiome also contains the virome and fungiome. The convergence of the microbiome and metabolic window omics is an area with potential for optimization, particularly during important developmental phases. Potential modifications include taking probiotic supplements, reducing antibiotic exposure, and using the microbiome as a biomarker for precision treatment. These pathways need to be investigated further, and while there are many extensive multi-omics studies in the adult literature, pediatric data is limited. To examine the makeup and impact of changes in the gut microbiome, clinical trials, particularly in large cohorts of healthy children and children with various disorders, are required. Moreover, geography and food culture have an impact on the gut flora in children. Depending on the research subjects chosen and the research methodologies employed, various countries and regions may reach different findings. Table 4 illustrates the clinical studies on intestinal microbiota and child growth in this review based on the cities/countries where the research was conducted, as well as the research methodology used, characteristics of the included populations, and main conclusions. Currently, the countries and regions undertaking study on this topic are primarily centered in Europe (28–30, 37, 38, 45, 47, 54, 57), the United States (33, 43, 46, 48, 51, 56), and China (39, 44, 53, 62, 76), with Africans, Bangladeshis, Europeans, and Chinese being the primary research subjects recruited. The primary areas of study are premature birth, intrauterine growth retardation, environmental sanitation, malnutrition, and so on. Chinese researchers are more interested in precocious puberty. According to the existing research literature, the association between gut microbiota and adolescent linear growth has received insufficient attention, and clinical research publications are limited. Furthermore, a better understanding of the relationships between the diverse microbiota and their components (bacteria, viruses, and fungi) is required (12). However, the mechanisms by which factors like maternal diet during pregnancy influence the microbiome of the offspring remain unknown. The vast majority of studies have been conducted in rodent models, with only a few mechanistic research studies conducted in humans. As a result, more validation is required before proceeding to clinical studies. Furthermore, there can be significant inter-individual variation in microbiota composition and drug responsiveness, meaning that conventional microbiota treatment may not be appropriate for every stunted patient. In phenotypically healthy populations, the use of multi-omics approaches such as metagenomics, metatranscriptomics, metaproteomics, and metabolomics aids in the discovery of microbial signals of growth retardation (2). Our present understanding of the gut microbiome is based on phylogenetic makeup (16S rRNA sequencing) or functional capacity (shotgun sequencing and identification of genes involved in metabolic pathways). Neither method fully captures trends in molecular crosstalk (caused by metabolites, antigens, signaling molecules, immunomodulators, and hormones) between the host and bacteria, resulting in systemic impacts on metabolism and the immune system. By including transcriptomics and metabolomics in the study of the dystrophic microbiota, researchers will gain a better understanding of how imbalances emerge. The majority of existing epidemiologic studies of malnutrition and gut microbiota are based on associations or correlations, making it impossible to determine the temporal order of these connections. To some extent, these problems can be addressed through longitudinal birth cohort studies of children or animal research. However, new and inventive ways are required to overcome these difficulties (12). With increased awareness and understanding comes the prospect of novel pharmaceutical targets and avenues for treating these diseases and promoting human health at all stages and ages (109).

**Table 4:** Clinacal studies that characterized gut microbiota associated with children's growth.

Regions and countries	Unfavorable conditions for children's growth	Study participants and age^a^	No.	Study's key findings	Methods	Ref.
EUROPE: Finland, Norway, Italy, Netherlands, Switzerland, France, Czech Republic, and Spain	Maternal overweight	Pregnant women 24 GW; m1 and m6; women 4 days after delivery and infants −y2	46; 42; 169	Bacteroidetes-dominated GMC in mid-pregnancy is associated with increased GWG and reduced alpha diversity (28). The composition and development of infant gut microbiota are influenced by BMI, weight, and weight gain of mothers during pregnancy (29). Maternal OW/OB was associated with lower maternal alpha diversity. Maternal pre-pregnancy OW/OB and excessive GWG were associated with taxonomic differences in the maternal gut microbiota (30).	16S rRNA sequencing	(28–30)
	Preterm birth	Preterm neonates (<35 GW), full-term neonates d3–d4; preterm neonates (≤30 GW)	80; 23	Preterm neonates exhibited significantly lower gut microbiota alpha diversity and distinct beta diversity clustering compared to term neonates (37). An increase in *α*-diversity values and a consequent fall in *Lactobacillus* in vaginal environment could be associated to a higher risk of spontaneous preterm birth (38).	16S rRNA sequencing	(37, 38)
	Poor infant hygiene	Infants −y6	12,422	Neonatal antibiotic exposure is linked to long-term gut microbiome disruption and may result in reduced growth in boys during the first 6 years of life, but antibiotic use later in childhood is linked to an increase in BMI (54).	16S rRNA sequencing	(54)
	Eating patterns and nutritional status	−m6; m3–m12	108; 98	After birth, colonization by *Bifidobacterium*, *Lactobacillus*, and *Bacteroides* species was modified by manner of delivery, type of feeding, and the presence of siblings, with differences observed at the species and temporal levels (45). Human milk oligosaccharides supplementation increased the number of newborns with fecal community types *Bifidobacteriaceae* in abundance (47).	16S rRNA sequencing	(45, 47)
	Gut inflamation	Stunted children y2–y5	57	The vast majority of the stunted children showed small intestinal bacterial overgrowth dominated by bacteria that normally reside in the oropharyngeal cavity (57).	16S rRNA sequencing; semiquantitative culture	(57)
United States	Preterm birth	Pregnant mothers in the early third trimester or intrapartum	163	Independent of maternal BMI, a high-fat maternal diet is associated with significant changes in the newborn gut microbiota at birth that last for 4–6 weeks (33).	16S rRNA sequencing	(33)
	SGA	Neonates with SGA, within 2 h after delivery	40	SGA patients had a considerably higher abundance of *Neisseriaceae* (43).	16S rRNA sequencing	(43)
	Eating patterns and nutritional status	Preterm infants −y5; stunted infant 6m	160; 25	Postnatal gut microbial colonization, a controllable factor, was linked to preterm infants’ childhood growth (46). Sialylated milk oligosaccharides promote microbiota-dependent growth in models of infant undernutrition (48).	16S rRNA sequencing, Microbial RNA-Seq, Mass spectroscopy	(46, 48)
	Poor infant hygiene	Bangladeshi y2	90	SIBO is connected with linear growth failure and inadequate sanitation, regardless of recent or frequent diarrheal illness. SIBO has been linked to intestinal inflammation but not to increased permeability or systemic inflammation (51).	Enzyme-linked immunosorbent assay	(51)
	Gut inflamation	Peruvian infants m5–m12	78	Stunting in infants was preceded by an increase in indicators of enterocyte turnover and variations in fecal microbiota, and it was linked to higher levels of systemic inflammatory markers (56).	16S rRNA sequencing	(56)
China	Preterm birth	Preterm infants −y1	166	Determine the dynamic changing features of newborns’ gut microbiomes at various gestational ages (39).	16S rRNA sequencing	(39)
	SGA	Term SGA and AGA neonates −7d	162	The gut microbial diversity of term SGA infants was significantly lower in the first week of life than that of term AGA infants (44).	16S rRNA sequencing	(44)
	Antibiotic exposure	Mother-child pairs m2–m6	295	Intrauterine antibiotic exposure can have an effect on newborn growth, and the neonatal gut flora may be involved (53).	16S rRNA sequencing	(53)
	Psychiatric diseases	Children with ADHD y6–y12	17	Differences in gut microbiota composition in ADHD participants may contribute to brain-gut axis abnormalities and impact neurotransmitter levels, which may contribute to ADHD symptoms (62).	Shotgun metagenomics sequencing	(62)
	Precocious puberty	Girls with ICPP y6–y8	48	The ICPP girls’ intestinal flora is diversified, similar to that of obese children, and rich in short-chain fatty acid-producing intestinal flora (76).	16S rRNA sequencing	(76)
México	Obesity	OW/OB children y6–y12	46	The synergy between nutrition and the composition of children's gut microbiota may be a factor in the development of juvenile obesity and its complications (71).	Metagenome shotgun sequencing	(71)

GWG, gestational weight gain; GMC, gut microbiota composition; OW/OB, overweight/obese; SIBO, small intestine bacterial overgrowth; SGA, small for gestational age; AGA, appropriate for gestational age; BMI, body mass index; ASD, autism spectrum disorder; ICPP, idiopathic central precocious puberty.

^a^ y, year of age; m, month of age; w, week of age; d, days of age.

## Limitations

The purpose of this study is to review the establishment and alterations of the gut microbiota in children at various phases of development, as well as the impact on children's linear growth. We mainly talked about the association between growth and development disorders at various stages and changes in gut flora, but we also briefly discussed the connection between gut flora and nutrition, neurophysiology, endocrine, and immune inflammation in children at various growth and development stages. They have complicated interactions with each other. This review mostly includes cohort studies (26, 28, 30, 39, 41, 44, 45, 48, 53, 54, 56, 59), cross-sectional studies (31, 37, 46, 51, 71, 76, 78), case-control studies (29, 47, 55, 57, 62), and some animal studies focusing on particular mechanisms (48, 87, 88). Although the data supports a link between intestinal flora and linear development in children, our review has limitations that prohibit us from fully verifying the causal relationship between gut flora and linear development in children. First, there is a bidirectional association between changes in the gut microbiota and the risk/resilience of children to linear growth disorders, making it difficult to separate between cause and effect. The cohort and cross-sectional studies included in this review were limited to account for the complexities of gut microbiome affects on linear growth in humans due to sample size and study scope limitations. Second, to determine the causal link between the gut microbiota and linear growth, long-term clinical controlled trials with large sample sizes are required. However, due to the limits of currently available 16S RNA sequencing or metagenomics-based research methodologies, determining which species or groups of gut bacteria play a key role in addressing growth problems, even in animal studies, is difficult. As a result, the clinical research included in this review primarily used commercial probiotic or prebiotic supplements as treatment options, and the findings of these studies do not yet indicate which strains are relevant for resolving growth deficiency in which situations. Furthermore, the goal of this literature review is to provide readers with up-to-date and thorough research advances on the association between gut microbiota and linear growth in children. Although relevant studies are categorised and described, it is a general literature review with limits in how to evaluate a set of studies and make appropriate recommendations, which is inferior to the evaluation effect of a systematic review. In addition, we included research material on systematic reviews (63), meta-analyses (73), and biological data mining investigations (70) in this review. However, because of the scarcity of existing study data and a small number of research projects engaged, these studies have limited interpretation of the association between gut microbiota and linear development in children. In short, due to the limitations of current research, our review can only attempt to educate readers of the most recent findings in key subject areas. When the relevant research literature is substantial, a systematic review study on this topic can be done in the future to compensate for the deficiencies of this review.
